# 4-Bromo-5-[(5,5-dimethyl-4,5-dihydro­isoxazol-3-yl)sulfonyl­meth­yl]-3-methyl-1-(2,2,2-trifluoro­ethyl)-1*H*-pyrazole

**DOI:** 10.1107/S1600536809040380

**Published:** 2009-10-10

**Authors:** Hong-Ju Ma, Qian-Fei Zhao, Xiang-Dong Mei, Jun Ning

**Affiliations:** a Key Laboratory of Pesticide Chemistry and Application, Ministry of Agriculture, Institute of Plant Protection, Chinese Academy of Agricultural Sciences, Beijing 100193, People’s Republic of China

## Abstract

In the title compound, C_12_H_15_BrF_3_N_3_O_3_S, which has potential herbicidal activity, the mol­ecule is twisted, as indicated by the C—S—C—C torsion angle of 67.86 (19)° for the atoms linking the ring systems. An intra­molecular C—H⋯F short contact occurs and inter­molecular C—H⋯O inter­actions link the mol­ecules in the crystal.

## Related literature

For background to pyrazoles and their pharmacological and pharmaceutical applications, see: Aiello *et al.* (2000 ▶); Hirai *et al.* (2002 ▶); Lahm *et al.* (2007 ▶); Meegalla *et al.* (2004 ▶); Ohno *et al.* (2004 ▶); Shiga *et al.* (2003 ▶); Sivaprasad *et al.* (2006 ▶); Vicentini *et al.* (2005 ▶); Waldrep *et al*. (1990 ▶). The trifluoro­methyl group is present in many biologically active pharmaceutical and agrochemical compounds, presumably due to its increased lipophilicity, electronegativity and relatively small size, see: Welch (1987 ▶).
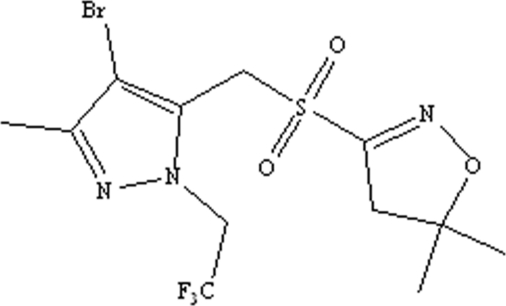


## Experimental

### 

#### Crystal data

C_12_H_15_BrF_3_N_3_O_3_S
*M* *_r_* = 418.24
Monoclinic, 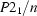

*a* = 16.127 (3) Å
*b* = 5.4356 (11) Å
*c* = 19.135 (4) Å
β = 106.85 (3)°
*V* = 1605.3 (6) Å^3^
*Z* = 4
Mo *K*α radiation
μ = 2.74 mm^−1^
*T* = 173 K
0.16 × 0.15 × 0.05 mm

#### Data collection

Rigaku MM007HF + CCD (Saturn724+) diffractometer
Absorption correction: numerical (*CrystalClear*; Rigaku, 2008 ▶) *T*_min_ = 0.668, *T*_max_ = 0.875
11131 measured reflections
3614 independent reflections
3353 reflections with *I* > 2σ(*I*)
*R*_int_ = 0.040

#### Refinement

*R*[*F* ^2^ > 2σ(*F* ^2^)] = 0.034
*wR*(*F* ^2^) = 0.093
*S* = 1.14
3614 reflections
211 parameters
H-atom parameters constrained
Δρ_max_ = 0.44 e Å^−3^
Δρ_min_ = −0.50 e Å^−3^

### 

Data collection: *CrystalClear* (Rigaku, 2008 ▶); cell refinement: *CrystalClear*; data reduction: *CrystalClear*; program(s) used to solve structure: *SHELXS97* (Sheldrick, 2008 ▶); program(s) used to refine structure: *SHELXL97* (Sheldrick, 2008 ▶); molecular graphics: *Mercury* (Macrae *et al.*, 2006 ▶); software used to prepare material for publication: *SHELXL97*.

## Supplementary Material

Crystal structure: contains datablocks I, global. DOI: 10.1107/S1600536809040380/hb5125sup1.cif

Structure factors: contains datablocks I. DOI: 10.1107/S1600536809040380/hb5125Isup2.hkl

Additional supplementary materials:  crystallographic information; 3D view; checkCIF report

## Figures and Tables

**Table 1:** Hydrogen-bond geometry (Å, °)

*D*—H⋯*A*	*D*—H	H⋯*A*	*D*⋯*A*	*D*—H⋯*A*
C7—H7*A*⋯F2	0.99	2.44	3.229 (3)	137
C5—H5*A*⋯O1^i^	0.99	2.30	3.131 (3)	141
C7—H7*B*⋯O2^ii^	0.99	2.29	3.271 (3)	169

Symmetry codes: (i) 
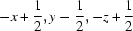
; (ii) 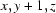.

**Fig. 1.**
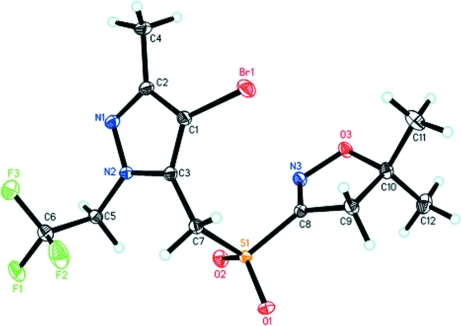
The molecular structure of the title compound, showing 50° probability displacement ellipsoids and the atom-numbering scheme.

## supplementary crystallographic information

### Comment

Pyrazole derivatives represent one of the most important classes of organic heterocyclic compounds, possessing a wide spectrum of biological activities in agrochemicals such as insecticidal (Lahm *et al.*, 2007; Meegalla *et al*., 2004; Shiga *et al.*, 2003), fungicidal (Aiello *et al.*, 2000; Sivaprasad *et al.*, 2006), herbicidal (Ohno *et al.*, 2004; Vicentini *et al.*, 2005; Waldrep *et al.*, 1990) activities. Some pyrazole derivatives are in use as herbicides such as pyrazolate, pyrazoxyfen, benzofenap, pyraflufen-ethyl, fluazolate and pyrazosulfuron-ethyl (Hirai *et al.*,2002). The trifluoromethyl moiety is particularly encountered in many biologically active pharmaceutical and agrochemical compounds presumably due to its increased lipophilicity, electronegativity and relatively small size (Welch 1987). Recently, we introduced trifluoromethyl to forming a novel title compound (I) with high herbicidal activity that has not been reported in literatures. The crystal structure of the title compound is shown in Fig. 1.

### Experimental

4-Bromo-5-[(5,5-dimethyl-4,5-dihydroisoxazol-3-yl)sulfonylmethyl]-3-methyl-1-(2,2,2-trifluoroethyl)-1*H*-pyrazole (0.2 g) was dissolved in acetone (50 ml) at room temperature. Colourless crystals of the title compound (I) were obtained through slow evaporation after two weeks.

### Refinement

The H atoms were placed at calculated positions, with C—H=0.93–0.98 Å, and were included in the refinement in the riding model approximation with *U*iso(H) set to 1.2 - 1.5*U*eq(C).

### Crystal data

**Table:**

C_12_H_15_BrF_3_N_3_O_3_S	*F*(000) = 840
*M**_r_* = 418.24	*D*_x_ = 1.730 Mg m^−^^3^
Monoclinic, *P*2_1_/*n*	Mo *K*α radiation, λ = 0.71073 Å
Hall symbol: -P 2yn	Cell parameters from 5465 reflections
*a* = 16.127 (3) Å	θ = 1.5–27.5°
*b* = 5.4356 (11) Å	µ = 2.74 mm^−^^1^
*c* = 19.135 (4) Å	*T* = 173 K
β = 106.85 (3)°	Slab, colourless
*V* = 1605.3 (6) Å^3^	0.16 × 0.15 × 0.05 mm
*Z* = 4	

### Data collection

**Table:**

Rigaku MM007HF + CCD (Saturn724+) diffractometer	3614 independent reflections
Radiation source: Rotating Anode	3353 reflections with *I* > 2σ(*I*)
Confocal	*R*_int_ = 0.040
Detector resolution: 28.5714 pixels mm^-1^	θ_max_ = 27.5°, θ_min_ = 1.5°
ω scans at fixed χ = 45°	*h* = −20→18
Absorption correction: numerical (*CrystalClear*; Rigaku, 2008)	*k* = −7→6
*T*_min_ = 0.668, *T*_max_ = 0.875	*l* = −21→24
11131 measured reflections	

### Refinement

**Table:**

Refinement on *F*^2^	Primary atom site location: structure-invariant direct methods
Least-squares matrix: full	Secondary atom site location: difference Fourier map
*R*[*F*^2^ > 2σ(*F*^2^)] = 0.034	Hydrogen site location: inferred from neighbouring sites
*wR*(*F*^2^) = 0.093	H-atom parameters constrained
*S* = 1.14	*w* = 1/[σ^2^(*F*_o_^2^) + (0.0399*P*)^2^ + 1.0315*P*] where *P* = (*F*_o_^2^ + 2*F*_c_^2^)/3
3614 reflections	(Δ/σ)_max_ = 0.001
211 parameters	Δρ_max_ = 0.44 e Å^−^^3^
0 restraints	Δρ_min_ = −0.50 e Å^−^^3^

### Special details

**Table:**

Geometry. All e.s.d.'s (except the e.s.d. in the dihedral angle between two l.s. planes) are estimated using the full covariance matrix. The cell e.s.d.'s are taken into account individually in the estimation of e.s.d.'s in distances, angles and torsion angles; correlations between e.s.d.'s in cell parameters are only used when they are defined by crystal symmetry. An approximate (isotropic) treatment of cell e.s.d.'s is used for estimating e.s.d.'s involving l.s. planes.
Refinement. Refinement of *F*^2^ against ALL reflections. The weighted *R*-factor *wR* and goodness of fit *S* are based on *F*^2^, conventional *R*-factors *R* are based on *F*, with *F* set to zero for negative *F*^2^. The threshold expression of *F*^2^ > σ(*F*^2^) is used only for calculating *R*-factors(gt) *etc*. and is not relevant to the choice of reflections for refinement. *R*-factors based on *F*^2^ are statistically about twice as large as those based on *F*, and *R*- factors based on ALL data will be even larger.

### Fractional atomic coordinates and isotropic or equivalent isotropic displacement parameters (Å^2^)

**Table:**

	*x*	*y*	*z*	*U*_iso_*/*U*_eq_	
Br1	0.445686 (15)	0.94374 (5)	0.090555 (14)	0.03063 (10)	
S1	0.36847 (3)	0.46277 (11)	0.24140 (3)	0.01880 (13)	
F1	0.05557 (10)	0.1917 (4)	0.03774 (10)	0.0506 (5)	
F2	0.10792 (11)	0.5508 (4)	0.06883 (11)	0.0533 (5)	
F3	0.09624 (11)	0.4150 (4)	−0.03880 (9)	0.0446 (5)	
O1	0.34862 (11)	0.5488 (4)	0.30560 (9)	0.0302 (4)	
O2	0.35701 (10)	0.2071 (3)	0.22232 (9)	0.0278 (4)	
O3	0.60544 (10)	0.5023 (3)	0.23928 (9)	0.0247 (4)	
N1	0.29249 (12)	0.3877 (4)	−0.01482 (10)	0.0245 (4)	
N2	0.27115 (12)	0.4000 (4)	0.04901 (10)	0.0202 (4)	
N3	0.52217 (12)	0.4076 (4)	0.22290 (11)	0.0218 (4)	
C1	0.36847 (14)	0.6842 (4)	0.05707 (12)	0.0215 (5)	
C2	0.35194 (15)	0.5606 (5)	−0.01018 (12)	0.0233 (5)	
C3	0.31611 (14)	0.5796 (4)	0.09432 (12)	0.0190 (4)	
C4	0.39090 (17)	0.6042 (6)	−0.07062 (14)	0.0352 (6)	
H4A	0.3649	0.4916	−0.1110	0.053*	
H4B	0.4535	0.5753	−0.0531	0.053*	
H4C	0.3800	0.7745	−0.0876	0.053*	
C5	0.20495 (14)	0.2363 (5)	0.05891 (12)	0.0235 (5)	
H5A	0.2172	0.1966	0.1114	0.028*	
H5B	0.2064	0.0809	0.0323	0.028*	
C6	0.11590 (16)	0.3492 (5)	0.03153 (14)	0.0306 (6)	
C7	0.30517 (14)	0.6434 (5)	0.16676 (12)	0.0203 (4)	
H7A	0.2433	0.6247	0.1641	0.024*	
H7B	0.3206	0.8188	0.1770	0.024*	
C8	0.47706 (14)	0.5475 (4)	0.25141 (12)	0.0180 (4)	
C9	0.52301 (14)	0.7679 (5)	0.29165 (13)	0.0242 (5)	
H9A	0.5152	0.7824	0.3409	0.029*	
H9B	0.5041	0.9221	0.2641	0.029*	
C10	0.61659 (14)	0.6997 (4)	0.29502 (13)	0.0221 (5)	
C11	0.66758 (17)	0.9027 (5)	0.27253 (17)	0.0347 (6)	
H11A	0.7229	0.8370	0.2691	0.052*	
H11B	0.6786	1.0347	0.3090	0.052*	
H11C	0.6343	0.9682	0.2249	0.052*	
C12	0.66479 (18)	0.5845 (5)	0.36726 (15)	0.0359 (6)	
H12A	0.7202	0.5188	0.3643	0.054*	
H12B	0.6300	0.4508	0.3786	0.054*	
H12C	0.6754	0.7093	0.4058	0.054*	

### Atomic displacement parameters (Å^2^)

**Table:**

	*U*^11^	*U*^22^	*U*^33^	*U*^12^	*U*^13^	*U*^23^
Br1	0.02683 (15)	0.02743 (17)	0.03790 (17)	−0.00740 (9)	0.00979 (11)	0.00368 (10)
S1	0.0184 (3)	0.0222 (3)	0.0175 (3)	−0.00311 (19)	0.0078 (2)	−0.0020 (2)
F1	0.0246 (8)	0.0738 (14)	0.0495 (10)	−0.0205 (8)	0.0044 (7)	0.0103 (9)
F2	0.0288 (9)	0.0612 (13)	0.0659 (13)	0.0097 (8)	0.0074 (8)	−0.0243 (10)
F3	0.0334 (9)	0.0640 (13)	0.0331 (9)	0.0067 (8)	0.0043 (7)	0.0170 (8)
O1	0.0277 (9)	0.0473 (12)	0.0192 (8)	−0.0046 (8)	0.0126 (7)	−0.0057 (8)
O2	0.0285 (9)	0.0204 (9)	0.0328 (9)	−0.0056 (7)	0.0065 (7)	0.0001 (7)
O3	0.0180 (8)	0.0272 (9)	0.0307 (9)	−0.0019 (6)	0.0098 (7)	−0.0084 (7)
N1	0.0237 (10)	0.0334 (12)	0.0174 (9)	0.0014 (8)	0.0076 (8)	−0.0002 (8)
N2	0.0185 (9)	0.0257 (10)	0.0173 (9)	−0.0007 (7)	0.0065 (7)	−0.0004 (8)
N3	0.0187 (9)	0.0221 (10)	0.0251 (10)	−0.0013 (7)	0.0070 (8)	−0.0031 (8)
C1	0.0183 (10)	0.0239 (12)	0.0220 (11)	0.0001 (8)	0.0055 (8)	0.0036 (9)
C2	0.0204 (11)	0.0320 (14)	0.0185 (11)	0.0040 (9)	0.0072 (9)	0.0041 (9)
C3	0.0180 (10)	0.0201 (11)	0.0187 (10)	0.0014 (8)	0.0047 (8)	0.0009 (8)
C4	0.0289 (13)	0.0569 (19)	0.0236 (13)	−0.0009 (12)	0.0133 (10)	0.0072 (12)
C5	0.0231 (11)	0.0254 (12)	0.0219 (11)	−0.0066 (9)	0.0066 (9)	0.0003 (9)
C6	0.0242 (12)	0.0373 (15)	0.0295 (13)	−0.0075 (11)	0.0067 (10)	0.0009 (11)
C7	0.0190 (10)	0.0207 (12)	0.0217 (11)	0.0008 (8)	0.0067 (8)	−0.0028 (9)
C8	0.0181 (10)	0.0189 (11)	0.0170 (10)	−0.0008 (8)	0.0049 (8)	−0.0015 (8)
C9	0.0199 (11)	0.0235 (12)	0.0298 (12)	−0.0028 (9)	0.0083 (9)	−0.0064 (10)
C10	0.0208 (11)	0.0183 (12)	0.0261 (12)	−0.0002 (8)	0.0053 (9)	−0.0037 (9)
C11	0.0256 (13)	0.0256 (14)	0.0546 (18)	−0.0013 (10)	0.0141 (12)	0.0048 (12)
C12	0.0350 (14)	0.0383 (16)	0.0277 (14)	0.0000 (11)	−0.0014 (11)	0.0005 (11)

### Geometric parameters (Å, °)

**Table:**

Br1—C1	1.868 (2)	C4—H4B	0.9800
S1—O1	1.4346 (17)	C4—H4C	0.9800
S1—O2	1.4351 (18)	C5—C6	1.509 (3)
S1—C8	1.767 (2)	C5—H5A	0.9900
S1—C7	1.789 (2)	C5—H5B	0.9900
F1—C6	1.327 (3)	C7—H7A	0.9900
F2—C6	1.334 (3)	C7—H7B	0.9900
F3—C6	1.339 (3)	C8—C9	1.499 (3)
O3—N3	1.387 (2)	C9—C10	1.537 (3)
O3—C10	1.487 (3)	C9—H9A	0.9900
N1—C2	1.327 (3)	C9—H9B	0.9900
N1—N2	1.363 (3)	C10—C12	1.511 (3)
N2—C3	1.366 (3)	C10—C11	1.512 (3)
N2—C5	1.444 (3)	C11—H11A	0.9800
N3—C8	1.279 (3)	C11—H11B	0.9800
C1—C3	1.376 (3)	C11—H11C	0.9800
C1—C2	1.407 (3)	C12—H12A	0.9800
C2—C4	1.487 (3)	C12—H12B	0.9800
C3—C7	1.488 (3)	C12—H12C	0.9800
C4—H4A	0.9800		
			
O1—S1—O2	119.28 (11)	F1—C6—C5	110.8 (2)
O1—S1—C8	106.41 (11)	F2—C6—C5	112.2 (2)
O2—S1—C8	109.30 (10)	F3—C6—C5	112.5 (2)
O1—S1—C7	106.71 (11)	C3—C7—S1	114.91 (16)
O2—S1—C7	109.08 (11)	C3—C7—H7A	108.5
C8—S1—C7	105.17 (11)	S1—C7—H7A	108.5
N3—O3—C10	109.76 (16)	C3—C7—H7B	108.5
C2—N1—N2	105.69 (19)	S1—C7—H7B	108.5
N1—N2—C3	112.23 (19)	H7A—C7—H7B	107.5
N1—N2—C5	118.40 (19)	N3—C8—C9	115.9 (2)
C3—N2—C5	129.33 (19)	N3—C8—S1	117.85 (17)
C8—N3—O3	108.43 (18)	C9—C8—S1	126.24 (17)
C3—C1—C2	107.1 (2)	C8—C9—C10	99.33 (18)
C3—C1—Br1	125.64 (18)	C8—C9—H9A	111.9
C2—C1—Br1	127.23 (18)	C10—C9—H9A	111.9
N1—C2—C1	109.9 (2)	C8—C9—H9B	111.9
N1—C2—C4	121.4 (2)	C10—C9—H9B	111.9
C1—C2—C4	128.7 (2)	H9A—C9—H9B	109.6
N2—C3—C1	105.0 (2)	O3—C10—C12	106.28 (19)
N2—C3—C7	125.0 (2)	O3—C10—C11	106.6 (2)
C1—C3—C7	130.0 (2)	C12—C10—C11	112.6 (2)
C2—C4—H4A	109.5	O3—C10—C9	103.34 (17)
C2—C4—H4B	109.5	C12—C10—C9	112.1 (2)
H4A—C4—H4B	109.5	C11—C10—C9	114.9 (2)
C2—C4—H4C	109.5	C10—C11—H11A	109.5
H4A—C4—H4C	109.5	C10—C11—H11B	109.5
H4B—C4—H4C	109.5	H11A—C11—H11B	109.5
N2—C5—C6	111.7 (2)	C10—C11—H11C	109.5
N2—C5—H5A	109.3	H11A—C11—H11C	109.5
C6—C5—H5A	109.3	H11B—C11—H11C	109.5
N2—C5—H5B	109.3	C10—C12—H12A	109.5
C6—C5—H5B	109.3	C10—C12—H12B	109.5
H5A—C5—H5B	107.9	H12A—C12—H12B	109.5
F1—C6—F2	107.2 (2)	C10—C12—H12C	109.5
F1—C6—F3	107.2 (2)	H12A—C12—H12C	109.5
F2—C6—F3	106.7 (2)	H12B—C12—H12C	109.5
			
C2—N1—N2—C3	0.2 (3)	N2—C3—C7—S1	86.0 (2)
C2—N1—N2—C5	178.1 (2)	C1—C3—C7—S1	−96.3 (3)
C10—O3—N3—C8	10.9 (2)	O1—S1—C7—C3	−179.38 (16)
N2—N1—C2—C1	−0.1 (3)	O2—S1—C7—C3	−49.28 (19)
N2—N1—C2—C4	−179.6 (2)	C8—S1—C7—C3	67.86 (19)
C3—C1—C2—N1	0.0 (3)	O3—N3—C8—C9	1.1 (3)
Br1—C1—C2—N1	−179.51 (17)	O3—N3—C8—S1	−177.89 (14)
C3—C1—C2—C4	179.4 (2)	O1—S1—C8—N3	148.46 (19)
Br1—C1—C2—C4	0.0 (4)	O2—S1—C8—N3	18.4 (2)
N1—N2—C3—C1	−0.2 (3)	C7—S1—C8—N3	−98.6 (2)
C5—N2—C3—C1	−177.8 (2)	O1—S1—C8—C9	−30.4 (2)
N1—N2—C3—C7	178.0 (2)	O2—S1—C8—C9	−160.43 (19)
C5—N2—C3—C7	0.4 (4)	C7—S1—C8—C9	82.6 (2)
C2—C1—C3—N2	0.2 (2)	N3—C8—C9—C10	−11.7 (3)
Br1—C1—C3—N2	179.65 (16)	S1—C8—C9—C10	167.21 (17)
C2—C1—C3—C7	−177.9 (2)	N3—O3—C10—C12	100.6 (2)
Br1—C1—C3—C7	1.6 (4)	N3—O3—C10—C11	−139.07 (19)
N1—N2—C5—C6	−89.7 (2)	N3—O3—C10—C9	−17.6 (2)
C3—N2—C5—C6	87.8 (3)	C8—C9—C10—O3	16.2 (2)
N2—C5—C6—F1	176.9 (2)	C8—C9—C10—C12	−97.8 (2)
N2—C5—C6—F2	−63.4 (3)	C8—C9—C10—C11	131.9 (2)
N2—C5—C6—F3	56.9 (3)		

### Hydrogen-bond geometry (Å, °)

**Table:**

*D*—H···*A*	*D*—H	H···*A*	*D*···*A*	*D*—H···*A*
C7—H7A···F2	0.99	2.44	3.229 (3)	137
C5—H5A···O1^i^	0.99	2.30	3.131 (3)	141
C7—H7B···O2^ii^	0.99	2.29	3.271 (3)	169

Symmetry codes: (i) −*x*+1/2, *y*−1/2, −*z*+1/2; (ii) *x*, *y*+1, *z*.
